# Altered Chemokine Signalling in Endothelial Progenitor Cells from Acute Ulcerative Colitis Patients

**DOI:** 10.1155/2015/843980

**Published:** 2015-02-08

**Authors:** L. De Toni, A. Di Nisio, S. Magagna, A. Michielan, M. Martinato, G. C. Sturniolo, R. D'Incà, C. Foresta, A. Garolla

**Affiliations:** ^1^Department of Medicine, Section of Endocrinology and Centre for Human Reproduction Patholgy, University of Padova, Via Giustiniani 2, 35128 Padova, Italy; ^2^Department of Surgical and Gastroenterological Sciences, Section of Gastroenterology, University of Padova, Via Giustiniani 2, 38128 Padova, Italy

## Abstract

Ulcerative colitis (UC) is a chronic, idiopathic, inflammatory bowel disease, characterized by alternating stages of clinically active and inactive disease. UC exhibits several inflammatory characteristics, including immune activation, leukocyte infiltration, and altered vascular density. In UC, many of the upregulated inflammatory cytokines are proangiogenic and are released by diverse cell populations, such as infiltrating immune cells and endothelial cells (EC). Increasing evidences suggest that neovascularisation may involve also endothelial progenitor cells (EPCs). In this study we evaluated EPCs recruitment and homing, assessed by CXCR4 expression, in both acute and remitting phase of UC. We report an overall decrease of EPCs in UC patients (controls = 97,94 ± 37,34 cells/mL; acute = 31,10 ± 25,38 cells/mL; remitting = 30,33 ± 19,02 cells/mL; *P* < 0.001 for both UC groups versus controls). Moreover CXCR4^+^-EPCs, committed to home in inflammatory conditions, were found to be reduced in acute UC patients compared to both remitting patients and controls (acute = 3,13 ± 4,61 cells/mL; controls = 20,12 ± 14,0; remitting = 19,47 ± 12,83; *P* < 0,001). Interestingly, we found that administration of anti-inflammatory drugs in acute UC is associated with an increase in circulating EPCs, suggesting that this therapy may exert a strong influence on the progenitor cells response to inflammatory processes.

## 1. Introduction

Ulcerative colitis (UC) is a chronic, idiopathic, inflammatory bowel disease (IBD), classically characterized by alternating stages of clinically active and inactive disease, a pattern seen in 80–90% of patients [1, 2]. According to population-based studies, an intermittent course of the disease occurs in 40–65% of patients after the first disease flare, whereas a continuous course of active disease may be seen in 5–10% of patients [3, 4]. Inflammation involves the rectum in a majority of patients (95%) and extends proximally in a continuous and circumferential fashion [5]. UC may involve the entire colorectum, thus termed pancolitis, or only parts of it. Clinical presentation can be limited to the rectum in cases of proctitis or may involve the sigmoid colon with or without descending colon in left-sided colitis. A few patients may develop limited terminal ileal involvement that can be difficult to differentiate from Crohn's ileocolitis. UC shares several inflammatory characteristics with other chronic immune disturbances including immune activation, leukocyte infiltration into tissues, and altered vascular density [6]. Mucosal inflammation is generally superficial, although patients with severe UC may develop transmural inflammation and deep colonic ulcerations that increase the risk of toxic megacolon. Severe symptoms are less commonly seen with left-sided colitis and proctitis. In particular, UC is characterized by periods of relapse and remission with flares of disease activity occurring spontaneously or provoked by certain aggravating factors such as intercurrent illness, antibiotic use, or nonadherence with medical therapy [7]. Many of the inflammatory cytokines that are upregulated in IBD are proangiogenic, the best examples being IL-17 (produced by invasive Th17 cells) and TNF-*α* produced by diverse cell populations, such as infiltrating immune cells (macrophages and monocytes) [8, 9] and the endothelial cells (EC) [10]. While available data suggest that angiogenesis and inflammation frequently occur together, evidence of the pathophysiologic relevance of angiogenesis in IBD is under thorough investigation [11–13]. In this regard, there is increasing evidence suggesting that neovascularisation may not solely be the result of angiogenesis, but may also involve endothelial progenitor cells (EPCs) [14, 15].

EPCs derive from the bone marrow, migrate into the peripheral circulation, and participate to endothelial repair and to neoangiogenesis through differentiation into mature EC [16–18]. EPCs' response to inflammatory chemokines and cytokines represents a crucial moment in triggering endothelial repair [19, 20]. In particular the CXCR4/SDF-1*α* axis plays an important role in the recruitment of EPCs to the sites of angiogenesis and in endothelial repair, but this mechanism is still unexplored in IBD [21, 22]. In a previous study we demonstrated that UC patients in the remitting phase (RUC) have a significant reduction of peripheral blood circulating EPCs, with respect to controls [15]. The aim of this study is to widen the knowledge on EPCs recruitment also in the acute phase of UC (AUC), with a focus on the homing of this cell population by the study of CXCR4/SDF-1*α* pathway.

## 2. Methods

### 2.1. Subjects

The investigation was conformed to the principles of the Declaration of Helsinki. The Institutional Ethics Committee approved the protocol, and all patients provided written informed consent.

A total of 53 consecutive UC male patients, 20 patients featured by AUC and 33 by RUC, were evaluated for the study. UC diagnosis was performed according to clinical, endoscopic, and histopathologic evaluation as previously described [23]. Criteria for diagnosis of AUC were Mayo score [24] of 6–12 points and endoscopy subscore ≥2. Diabetes, smoking, arterial hypertension, high homocysteine levels, body mass index higher than 25, and previous major cardiovascular events were considered as exclusion criteria, since they were previously associated with a reduction of circulating EPCs [25]. Patients receiving statins, anti-TNF-*α* (tumor necrosis factor-*α*) monoclonal antibody (Infliximab), or PDE5i (phosphodiesterase type 5 inhibitors) were also excluded [15]. Thirty-three nonsmoking and age-matched healthy males served as controls. Fasting peripheral blood samples were obtained from each participant, kept at room temperature, and processed within 2-3 hours from withdrawal.

### 2.2. Evaluation of EPC Number

Circulating EPCs were counted in peripheral blood as previously described [15, 26]. Briefly, 450 *μ*L of peripheral blood was incubated with biotin-conjugated monoclonal anti-human VEGFR2 antibody (KDR, Sigma-Aldrich, Milano, Italy) and washed with PBS (phosphate-buffered saline). Samples were then incubated with phycoerythrin-labeled (PE) monoclonal anti-human CD34 antibody (Becton Dickinson, Milano, Italy), APC- (allophycocyanin-) labeled monoclonal anti-human CD133 antibody (Miltenyi Biotec, Bergisch Gladbach, Germany), streptavidin-FITC (fluorescein isothiocyanate) (Sigma-Aldrich). Erythrocytes were lysed using the Pharmlyse buffer (Becton Dickinson) and centrifuged for 5 min at 1,800 g. Samples were finally resuspended in 400 *μ*L of PBS and analyzed using the FACSCalibur Flow Cytometer (Becton Dickinson, San Jose, CA). EPCs were considered as circulating mononuclear cells featured by a triple positive staining for CD34, CD133, and KDR (Supplemental Figure S1-D, available online at http://dx.doi.org/10.1155/2015/843980).

Detection of CXCR4^+^-EPCs was performed by the addition of Pe-Cy5-labeled anti-human CXCR4 antibody (Becton Dickinson) in the reaction mixture as previously described. During flow cytometry analysis, CXCR4^+^-EPCs were identified as EPCs with a further positive staining for CXCR4 (Supplemental Figure S1-E).

### 2.3. Statistical Analysis

Data analysis was performed using SPSS version 13.0 (SPSS Inc., Chicago, IL, USA). The results are expressed as means ± standard deviations (SD). Levene's test was used to test the homogeneity of variance among groups prior to data analysis. If homogeneity of variance assumption was violated, Welch test was performed and the respective *P* value was reported. Differences between two groups were analysed using Student's *t*-test. Differences between three or more groups were analysed using Kruskal-Wallis post hoc test for ANOVA; Fisher's Least Significance Difference (LSD) adjustment for multiple comparisons of groups was applied to the pairwise comparisons of groups. When two or more dependent variables were tested simultaneously, a multivariate analysis of variance was performed to test for covariates effect on EPCs and CXCR4^+^-EPCs in AUC and RUC groups separately. Statistical significance was defined at the *P* < 0.05 level using 2-sided tests; highly statistical significance was defined for values of *P*< 0.01.

## 3. Results

The clinical characteristics of UC patients and age-matched controls are listed on Table 1. UC patients and controls did not differ for age (*P* = 0.40). Moreover, there was no difference in terms of age and duration of the disease between AUC and RUC patients (*P* = 0.57 and *P* = 0.32, resp.). Within AUC patients, the disease localization was equally distributed between cholic and pancholic disease (10/20 for both). In RUC patients, the localization was cholic in all subjects. Only one patient showed inflammation at both cholic and ileum sites. 15 out of 20 AUC patients and 6 out of 33 RUC patients were found to receive a multidrug therapy. 4 out of 33 RUC patients did not receive any drug treatment at the time of the enrolment.

**Figure 1:**
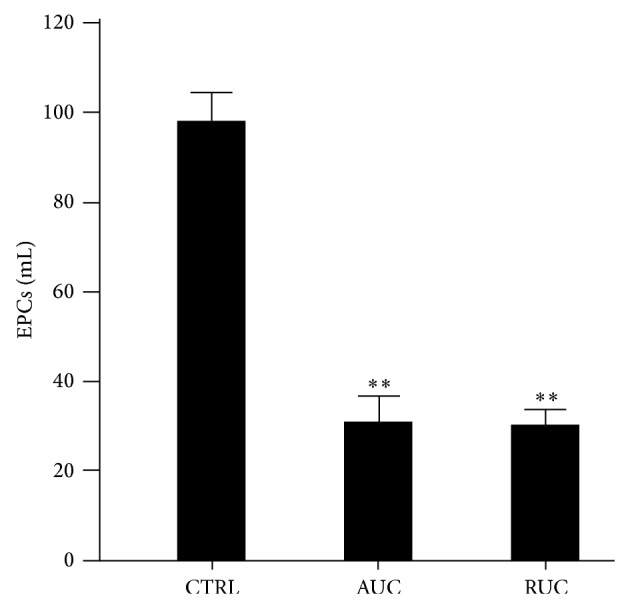
Circulating endothelial progenitor cells (EPCs) levels in each study group (CTRL = controls; AUC = active ulcerative colitis; RUC = remitting ulcerative colitis). Data are shown as means ± standard error means. Statistical significance was calculated with a univariate ANOVA and post hoc test for multiple comparisons. Statistical difference in AUC and RUC groups with respect to controls is indicated by ^**^(*P* < 0,001).

Figure 1 shows the number of circulating EPCs observed in AUC and RUC patients, compared to controls. UC patients had significantly lower circulating EPC levels than controls, in both acute and remitting phase (controls = 97,94 ± 37,34 cells/mL; AUC = 31,10 ± 25,38 cells/mL; RUC = 30,33 ± 19,02 cells/mL; *P* < 0.001 for both AUC and RUC versus controls).

**Figure 2:**
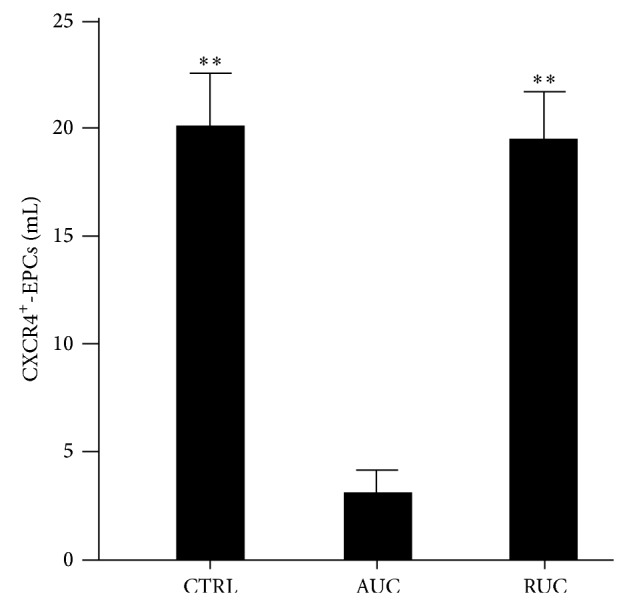
Circulating CXCR4-positive endothelial progenitor cells (CXCR4^+^-EPCs) levels in each study group (CTRL = controls; AUC = active ulcerative colitis; RUC = remitting ulcerative colitis). Data are shown as means ± standard error means. Statistical significance was calculated with a univariate ANOVA and post hoc test for multiple comparisons. Statistical difference in RUC patients and controls with respect to AUC group is indicated by ^**^(*P* < 0,001).

The evaluation of the EPC mobilization/homing, measured by the quantification of circulating CXCR4^+^-EPCs, was similar in RUC patients with respect to controls (*P* = 0.83). On the other hand, there was a significant reduction of this cell population in AUC patients compared to both controls and RUC patients (controls = 20,1 ± 14,0; AUC = 3,1 ± 4,6 cells/mL; RUC = 19,4 ± 12,8; *P* < 0.001 AUC versus both controls and RUC groups; Figure 2).

In AUC patients there was a trend towards reduction of EPCs with increasing age, even though not statistically significant (*P* = 0.06), whereas the duration of the disease and its localization did not affect EPC number. In RUC patients, circulating EPCs were not affected by age, duration of the disease, or localization (Table 2). Moreover, in AUC patients, the use of oral azathioprine, cyclosporine, or methotrexate did not correlate with EPC number (*P* = 0.48; *P* = 0.81 and *P* = 0.77, resp.), whereas the use of glucocorticoids led to a significant increase in circulating EPCs in these patients (*P* = 0.04; Supplemental Figure  S2). In addition, the use of oral and topic mesalazine, cyclosporine, and azathioprine did not affect EPCs in RUC patients (Table 2).

CXCR4^+^-EPCs in AUC patients showed a trend towards reduction with increasing age (*P* = 0.06), whereas the duration of the disease or localization did not influence circulating levels of this cell population (Table 2). In RUC patients, age, duration of the disease and localization did not affect CXCR4^+^-EPCs (Table 2).

Finally, in AUC patients, the use of oral glucocorticoids, azathioprine, cyclosporine, or methotrexate did not correlate with EPC number (Table 2). Administration of mesalazine in RUC patients did not affect CXCR4^+^-EPCs, and treatment with azathioprine led to a significant increase of this parameter (Supplemental Figure S3; Table 2), whereas the administration of cyclosporine was associated with a reduction of CXCR4^+^-EPCs (Supplemental Figure S4; Table 2).

## 4. Discussion

In this study we confirmed that UC patients in the remitting phase show a consistent reduction of the circulating levels of EPCs [15]. To our knowledge, this effect was not investigated in course of the acute phase of UC. Interestingly, we also observed a reduction of this circulating cell population in acute patients. In addition we documented a peculiar reduction of the CXCR4^+^-EPCs subtype, ascribed to homing competence, only in the acute phase, with respect to RUC patients and controls.

The microcirculation and its lining endothelium play a central role in the initiation and perpetuation of the inflammatory response, as well as in tissue remodelling during chronic inflammation. Investigation into the cellular and molecular mechanisms in human inflammatory bowel disease, such as ulcerative colitis, has demonstrated a central role for the intestinal microvascular endothelium in both normal mucosal immunity and the dysregulated chronic inflammation that characterizes IBD [10]. There is now increasing evidence to suggest that neovascularization in response to tissue damage may involve bone marrow-derived endothelial progenitor cells [14]. It has also been shown that the enhanced expression of CXCR4 in EPCs improves the chemotactic response of these cells versus stromal-derived factor 1. This chemokine, which is the specific agonist of CXCR4, is released during tissue damage and consequent inflammatory response [27], leading to the peripheral homing of progenitor cells in the endothelium unevenness contributing to endothelial repair [28].

There are several possible explanations for the observed reduction in the number of circulating EPCs in patients with UC. This reduction might be due to the consumption of circulating EPCs at the site of disease. As hypothesized by other authors, EPCs may be trapped in the inflamed intestinal vessels, resulting in a reduction of their numbers in the peripheral blood. In fact, this hypothesis is supported by recent observations of EPC enrichment in the inflamed rheumatoid arthritis joint [14, 29]. In alternative, Marlicz et al. [30] demonstrated that hematopoietic stem/progenitor cells, the putative precursors of EPCs, as well as pluripotent very small embryonic-like stem cells, are heavily mobilized into peripheral blood in patients with inflammatory conditions of chronic diseases. In this regard, the ability of haematopoietic progenitor cells to differentiate into EPCs may be impaired in patients with UC. If this were true, the factor responsible for this reduced ability of progenitor cells to differentiate may play a crucial role in the pathophysiology of UC [30]. In this scenario, the CXCR4/SDF-1*α* axis plays an important role in the recruitment of EPCs to the sites of angiogenesis and may be impaired in UC, as a consequent impairment of this pathway [21, 22]. In agreement with these considerations, during the acute phase of UC we observed a considerable alteration of EPC's competence in homing, as displayed by the strong decrease of CXCR4^+^-EPCs subtype in peripheral blood. This hypothesis is also supported by recent studies from Starzyńska et al. performed in patients suffering for pancreatic cancer [31]. In fact, authors demonstrated that pancreatic cancer was not associated with profound inflammatory process as UC and no significant difference in systemic circulation of bone marrow-derived EPC was observed in this kind of patients. Accordingly, the dependence of circulating levels of several bone marrow-derived stem cell populations, including EPCs, has been recently associated with systemic levels of multiple pro- and anti-inflammatory cytokines by an increasing number of studies [32].

Interestingly, we found that the administration of glucocorticoids in AUC is associated with an increase in circulating EPCs. Similarly, azathioprine seems to improve CXCR4^+^-EPCs levels in RUC. An overall improvement of EPC pattern, subsequent to administration of drugs with major anti-inflammatory effects, has been recently reported by Pirro et al. in a study evaluating patients affected by polymyalgia rheumatica [33]. Unfortunately, it is still unclear whether these modifications are related to drug-induced effects on EPCs or whether they are related to an impairment of clinical inflammatory status. Contrarily, the use of cyclosporine was associated with a significant reduction of CXCR4^+^-EPCs levels in RUC patients, suggesting a direct and inhibitory effect on this pathway. In this regard, in an in vitro study Davies et al. observed that cyclosporine prevented differentiation, inhibited proliferation and attenuated migration of EPCs [34]. Despite the low sample size and the unequal distribution of therapy assignment within UC groups, we could speculate that chronic phases of UC are characterized by strong reduction in the process of endothelial repair; moreover the use of drug therapies may exert a strong influence on the progenitor cells response to inflammatory process and homing. This aspect surely represents a challenging opportunity to widen the investigation on larger cohorts.

## Supplementary Material

Figure S1: Gating Strategy for Quantification of EPC and CXCR4+ EPC by Flow Cytometry Peripheral blood mononuclear cells were identified by morphological scatter-plot (plot A), gated in region R1 and evaluated for expression of CD34+ cells (plot B). CD34+ cells were then gated in region R2 and evaluated for the expression of CD133 (plot C). Only cells highly positive for CD133 were gated in region R3 and evaluated for KDR expression (plot D). Cells simultaneously positive for CD34, CD133 and KDR were identified as endothelial progenitor cells (EPCs) and assessed for CXCR4 expression (plot E).
Figure S2: Circulating endothelial progenitor cells (EPCs) levels in patients featured by active ul-cerative colitis (AUC) undergoing therapy with glucocorticoid drugs compared to untreated AUC patients. Data shown are the means ± standard error means. Statistical signifcance was calculated with a Student-t test. ∗ indicates statistical significance < 0,05.
Figure S3: Circulating CXCR4-positive endothelial progenitor cells (CXCR4+-EPCs) levels in the ab-sence or presence of therapy with Azathioprine (white bars and grey bars respectively) in patients featured by active ulcerative colitis (AUC) and by remitting ulcerative colitis (RUC). Data shown are the means ± standard error means. Statistical significance was calculated with a multivariate analysis of variance. Statistical difference in treated group with respect to untreated group within AUC and RUC patients is indicated by ∗ (P < 0,001).
Figure S4: Circulating CXCR4-positive endothelial progenitor cells (CXCR4+-EPCs) levels in the ab-sence or presence of therapy with Cyclosporine (white bars and grey bars respectively) in patients featured by active ulcerative colitis (AUC) and by remitting ulcerative colitis (RUC). Data shown are the means ± standard error means. Statistical significance was calculated with a multivariate analysis of variance. Statistical difference in treated group with respect to untreated group within AUC and RUC patients is indicated by ∗ (P < 0,001).

## Figures and Tables

**Table 1:** Clinical features of the study cohort.

	CTRL (*N* = 33)	AUC (*N* = 20)	RUC (*N* = 33)
Age (years)	40.4 ± 9.1 (23–50)	41.2 ± 11.1 (21–55)	42.7 ± 9.2 (25–57)
Duration of the disease (months)	—	87.0 ± 81.1 (0–228)	112.9 ± 97.3 (24–372)
Localization (number of cases)			
Cholic	—	10	33
Pancholic	—	10	—
Ileum	—	—	1
Therapy (number of cases)			
Glucocorticoids	—	14	—
Mesalazine	—	20	30
Azathioprine	—	3	2
Cyclosporine	—	1	2
Methotrexate	—	1	—

Means ± SD are reported. *N* indicates sample size. CTRL = control group; AUC = acute ulcerative colitis; RUC = remitting ulcerative colitis.

**Table 2:** Summary of *P* values for multivariate analysis of variance statistics in AUC and RUC patients.

	AUC (*N* = 20)		RUC (*N* = 33)	
	EPCs	CXCR4^+^-EPCs	EPCs	CXCR4^+^-EPCs
Age (years)	*P* = 0.06	*P* = 0.06	*P* = 0.23	*P* = 0.17
Duration of the disease	*P* = 0.65	*P* = 0.26	*P* = 0.40	*P* = 0.14
Localization				
Cholic				
Pancholic	*P* = 0.92	*P* = 0.46	*P* = 1.00	*P* = 1.00
Ileum				
Therapy				
Glucocorticoids	***P* = 0.04**	*P* = 0.23	—	—
Mesalazine	—	—	*P* = 0.90	*P* = 0.10
Azathioprine	*P* = 0.48	*P* = 0.80	*P* = 0.49	***P* = 0.01**
Cyclosporine	*P* = 0.81	*P* = 0.38	*P* = 0.51	***P* < 0.01**
Methotrexate	*P* = 0.77	*P* = 0.64	—	—

*N* indicates sample size. AUC = active ulcerative colitis; RUC = remitting ulcerative colitis. In bold are reported significant *P* values. Hyphen was used when statistical analysis was not performed because of missing data for that trait (see Table 1 for details).
