# Supplementary material for: Altered Chemokine Signalling in Endothelial Progenitor Cells from Acute Ulcerative Colitis Patients
Source: Gastroenterol Res Pract. 2015 Feb 8;2015:843980. doi: 10.1155/2015/843980 (PMC4337053; doi:10.1155/2015/843980)
Supplement: Supplementary file 1 — Figure S1: Gating Strategy for Quantification of EPC and CXCR4+ EPC by Flow Cytometry Peripheral blood mononuclear cells were identified by morphological scatter-plot (plot A), gated in region R1 and evaluated for expression of CD34+ cells (plot B). CD34+ cells were then gated in region R2 and evaluated for the expression of CD133 (plot C). Only cells highly positive for CD133 were gated in region R3 and evaluated for KDR expression (plot D). Cells simultaneously positive for CD34, CD133 and KDR were identified as endothelial progenitor cells (EPCs) and assessed for CXCR4 expression (plot E). Figure S2: Circulating endothelial progenitor cells (EPCs) levels in patients featured by active ul-cerative colitis (AUC) undergoing therapy with glucocorticoid drugs compared to untreated AUC patients. Data shown are the means ± standard error means. Statistical signifcance was calculated with a Student-t test. ∗ indicates statistical significance < 0,05. Figure S3: Circulating CXCR4-positive endothelial progenitor cells (CXCR4+-EPCs) levels in the ab-sence or presence of therapy with Azathioprine (white bars and grey bars respectively) in patients featured by active ulcerative colitis (AUC) and by remitting ulcerative colitis (RUC). Data shown are the means ± standard error means. Statistical significance was calculated with a multivariate analysis of variance. Statistical difference in treated group with respect to untreated group within AUC and RUC patients is indicated by ∗ (P < 0,001). Figure S4: Circulating CXCR4-positive endothelial progenitor cells (CXCR4+-EPCs) levels in the ab-sence or presence of therapy with Cyclosporine (white bars and grey bars respectively) in patients featured by active ulcerative colitis (AUC) and by remitting ulcerative colitis (RUC). Data shown are the means ± standard error means. Statistical significance was calculated with a multivariate analysis of variance. Statistical difference in treated group with respect to untreated group within [file 843980.f1.pdf]

LEGEND TO SUPPLEMENTAL FIGURES

Figure S1

Gating Strategy for Quantification of EPC and $CXCR4^+$ EPC by Flow Cytometry

Peripheral blood mononuclear cells were identified by morphological scatter-plot (plot A), gated in region R1 and evaluated for expression of CD34+ cells (plot B). CD34+ cells were then gated in region R2 and evaluated for the expression of CD133 (plot C). Only cells highly positive for CD133 were gated in region R3 and evaluated for KDR expression (plot D). Cells simultaneously positive for CD34, CD133 and KDR were identified as endothelial progenitor cells (EPCs) and assessed for CXCR4 expression (plot E).

Figure S2

Circulating endothelial progenitor cells (EPCs) levels in patients featured by active ulcerative colitis (AUC) undergoing therapy with glucocorticoid drugs compared to untreated AUC patients. Data shown are the means ± standard error means. Statistical significance was calculated with a Student-t test. * indicates statistical significance < 0,05.

Figure S3

Circulating CXCR4-positive endothelial progenitor cells ($CXCR4^+$-EPCs) levels in the absence or presence of therapy with Azathioprine (white bars and grey bars respectively) in patients featured by active ulcerative colitis (AUC) and by remitting ulcerative colitis (RUC). Data shown are the means ± standard error means. Statistical significance was calculated with a multivariate analysis of variance. Statistical difference in treated group with respect to untreated group within AUC and RUC patients is indicated by * ($P < 0{,}001$).

Figure S4

Circulating CXCR4-positive endothelial progenitor cells ($CXCR4^+$-EPCs) levels in the absence or presence of therapy with Cyclosporine (white bars and grey bars respectively) in patients featured by active ulcerative colitis (AUC) and by remitting ulcerative colitis (RUC). Data shown are the means ± standard error means. Statistical significance was calculated with a multivariate analysis of variance. Statistical difference in treated group with respect to untreated group within AUC and RUC patients is indicated by * ($P < 0{,}001$).

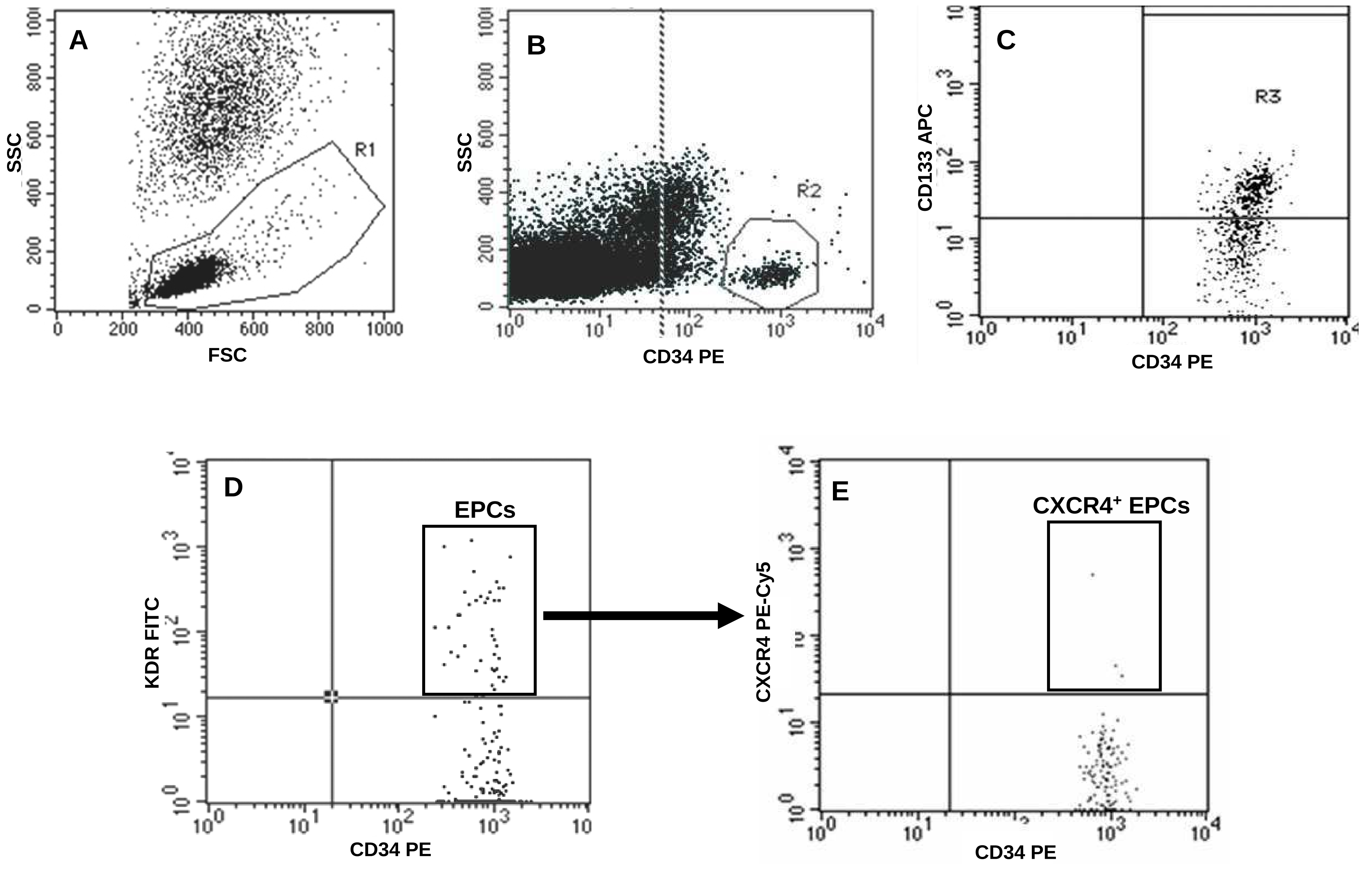
A
SSC
FSC
R1
B
SSC
CD34 PE
R2
C
CD133 APC
CD34 PE
R3
D
KDR FITC
CD34 PE
EPCs
E
CXCR4 PE-Cy5
CD34 PE
CXCR4+ EPCs

Figure S1

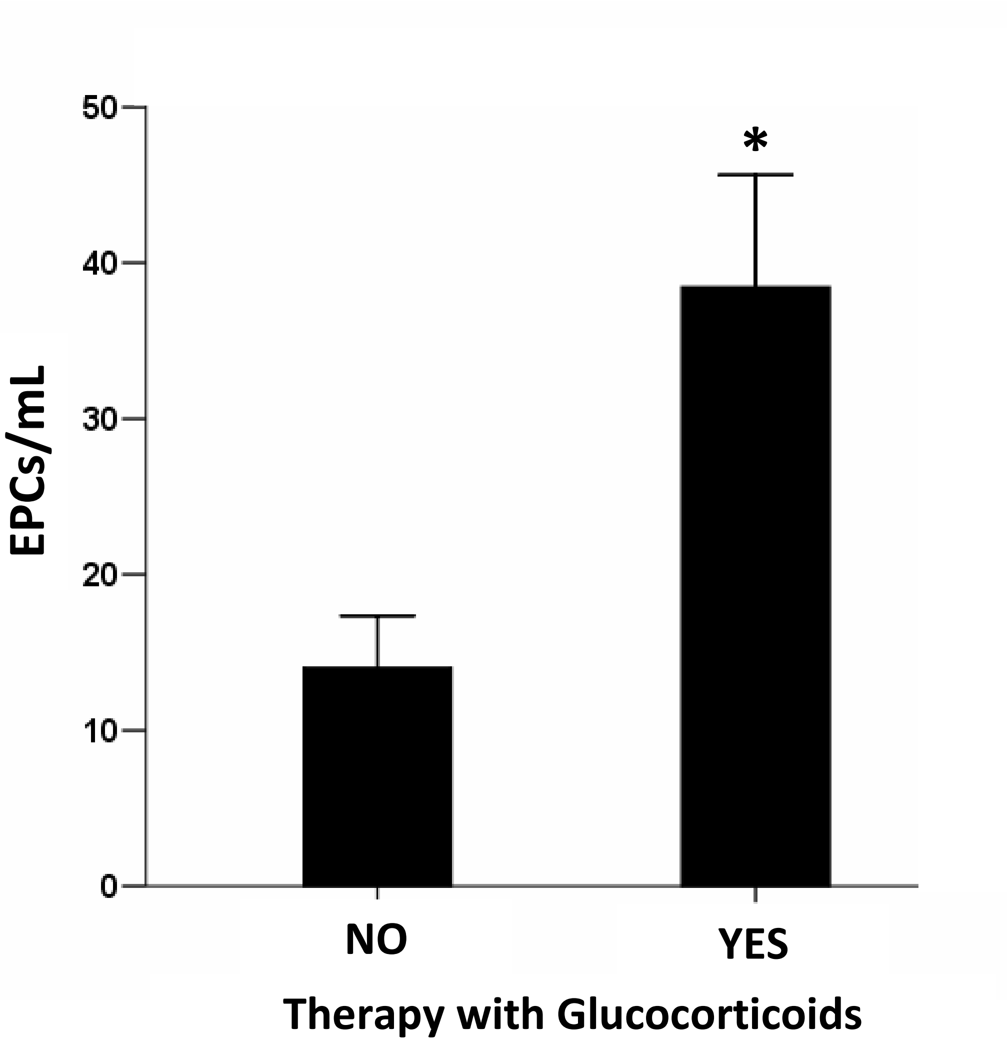

Figure S2

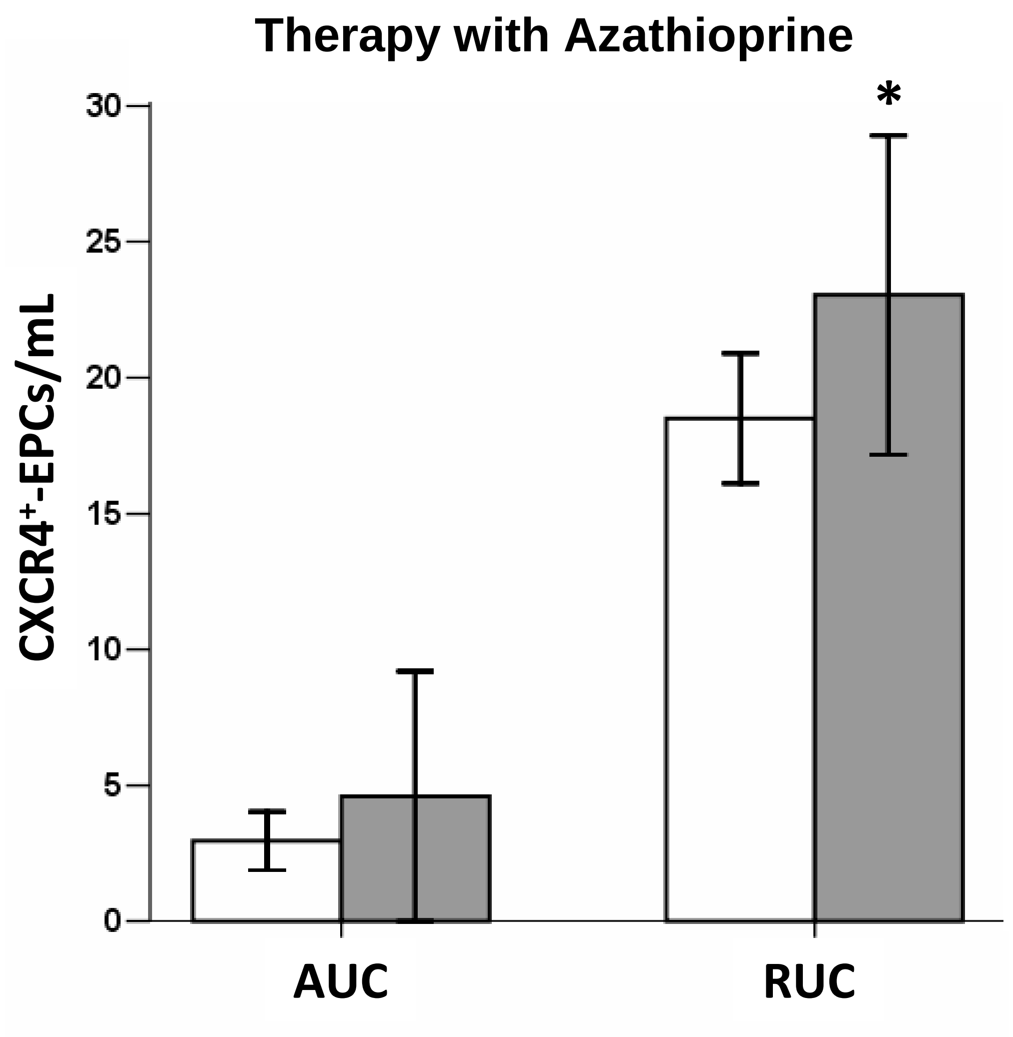
Therapy with Azathioprine
CXCR4⁺-EPCs/mL
0
5
10
15
20
25
30
*
AUC
RUC

Figure S3

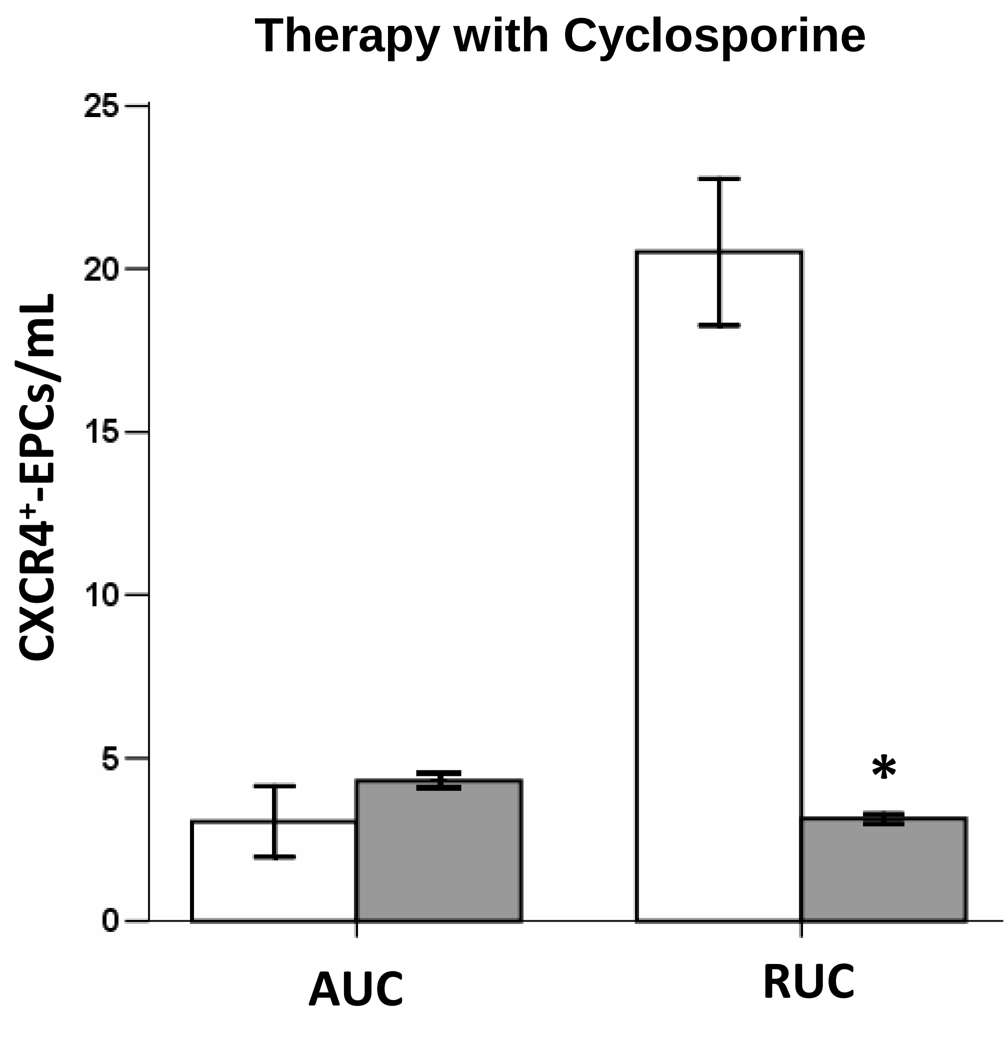
Therapy with Cyclosporine
CXCR4⁺-EPCs/mL
25
20
15
10
5
0
*
AUC
RUC

Figure S4
